# Durum wheat mutants with enhanced disease resistance to stripe rust show differential responses to other fungal diseases

**DOI:** 10.1007/s11032-025-01576-y

**Published:** 2025-06-16

**Authors:** China Lunde, Kyungyong Seong, Rakesh Kumar, Andrew Deatker, Bhavit Chhabra, Meinan Wang, Shivreet Kaur, Dina Raats, Christian Schudoma, Alex Schultink, Sarah Song, Ann Palayur, Cole Davies, William Cumbelich, Upinder Gill, Nidhi Rawat, Xianming Chen, Meriem Aoun, Christopher Mundt, Ksenia V. Krasileva

**Affiliations:** 1 Department of Plant and Microbial Biology, University of California, Berkeley, CA 94720 USA; 2 The Sainsbury Laboratory, Norwich Research Park, Norwich, NR4 7UH UK; 3 Department of Plant Sciences and Landscape Architecture, University of Maryland, College Park, MD 20742 USA; 4 Department of Plant Pathology, Washington State University, Pullman, WA 99164 USA; 5 Department of Entomology and Plant Pathology, North Carolina State University, Raleigh, NC 27695 USA; 6 Department of Plant Pathology, North Dakota State University, Fargo, ND 58108 USA; 7 United States Department of Agriculture, Agricultural Research Service, Wheat Health, Genetics, and Quality Research, Pullman, WA 99164 USA; 8 Department of Entomology and Plant Pathology, Oklahoma State University, Noble Research Center, Ardmore, OK 73401 USA; 9 Department of Botany and Plant Pathology, Oregon State University, Corvallis, OR 97331 USA; 10 Earlham Institute, Norwich Research Park, Norwich, NR4 7UZ UK; 11 Fortiphyte, Inc., Richmond, CA 94806 USA; 12 Crop Improvement and Genetics Research Unit, USDA-ARS, Western Regional Research Center, 800 Buchanan Street, Albany, CA 94710 USA

**Keywords:** Kronos, Yellow rust, *Puccinia striiformis*, Durum wheat, Fungal diseases, Resistance breeding, Spontaneous lesion

## Abstract

Introducing and characterizing variation through mutagenesis plus functional genomics can accelerate resistance breeding as well as our understanding of crop plant immunity. To reveal new germplasm resources for fungal disease resistance breeding in elite durum wheat, we challenged the diverse alleles in a sequenced and cataloged ethyl methanesulfonate mutagenized population of elite tetraploid wheat *Triticum turgidum* subsp. *durum* cv ‘Kronos’ with stripe rust. We screened 2,000 mutant lines and identified sixteen enhanced disease resistance (EDR) lines with persistent resistance to stripe rust over four years of field testing. To find broad-spectrum resistance, we challenged these lines with other major biotrophic and necrotrophic pathogens, including those causing Septoria tritici blotch, tan spot, Fusarium head blight and leaf rust. Enhanced resistance to multiple fungi was found in 13 of 16 EDR lines. Five EDR lines showed spontaneous lesion formation in the absence of pathogens, providing new mutant resources to study plant stress response in the absence of the confounding effects of pathogen infection. We mapped exome capture sequencing data of the EDR lines to a recently released long-read Kronos genome to aid in the identification of causal mutations. We located an EDR resistance locus to an 175 Mb interval on chromosome 1B. Importantly, these phenotypically characterized EDR lines are newly described durum germplasm coupled with improved functional genomics resources that are readily available for both wheat fungal resistance breeding and basic plant immunity research.

**Supplementary Information:**

The online version contains supplementary material available at 10.1007/s11032-025-01576-y.

## Introduction

Stripe rust, caused by the obligate biotrophic fungus, *Puccinia striiformis* f. sp. *tritici* (*Pst*), is an economically important disease that affects global wheat production. Over the last 50 years, the geographic range and severity of stripe rust has increased such that 88% of the world’s wheat is susceptible to rust. This disease manifests on wheat leaves and leads to severe yield losses, potentially amounting to a billion dollars a year globally (Beddow et al. 2015) when not controlled with fungicide applications (Chen 2014). Deploying genetic resistance is preferable for efficiency and reduced environmental impact but requires continual identification of new resistance alleles to combat pathogen evolution. Over the last decade, the severity of stripe rust has increased due to the emergence of new virulent races (Hubbard et al. 2015). Newer isolates of *Pst* are now found in wider geographic locations and have become more adapted to warmer and drier climates. Climate change is expected to facilitate the spread of *Pst* and exacerbate the vulnerability of wheat. Since 1.2 billion people depend upon wheat for their calories, it has been estimated that $32 M USD is a well-justified annual expenditure in the development and deployment of stripe rust-resistant varieties (Beddow et al. 2015).

Although commonly deployed, all-stage pathogen resistance is generally monogenic, dominant, pathogen race-specific, and quickly overcome by pathogen evolution but amenable to marker-assisted selection. In contrast, adult-stage resistance is often polygenic and more durable but requires careful phenotypic selection (Wang and Chen 2017). Stripe (yellow) rust resistance genes (designated *Yr*) have been central to developing commercial wheat varieties and are widely deployed (Marchal et al. 2018). Yet, mechanisms of stripe rust resistance vary substantially as evidenced by the variety of cloned *Yr* gene products (Shah et al. 2011; Moore et al. 2015; Wang and Chen 2017; Yuan et al. 2018; Marchal et al. 2018; Singh et al. 2023). Intracellular immune receptor *Yr5,* a BED-domain containing nucleotide-binding and leucine-rich repeat receptor (BED-NLR) (Marchal et al. 2018), and wheat tandem kinase *Yr15* (Klymiuk et al. 2018; Fahima et al. 2018) give complete all-stage resistance with no visible infection (Wang and Chen 2017). In contrast, mutating ABC transporter *Yr18* frequently causes leaf tip necrosis (Shah et al. 2011) and plants carrying hexose transporter *Yr46* often have leaves striped with chlorotic and necrotic tissue (Moore et al. 2015). The expression of these phenotypes is further influenced by host genetic background, the genotype of the *Pst* isolate and environmental conditions (Wang and Chen 2017). Currently, rampant spread of highly virulent *Pst* molecular group five (MG5) isolates, present in the United States, can only be restricted by the ubiquitously used broad-spectrum stripe rust-resistance genes *Yr*5 and *Yr15*, plus a few newly identified *Yr* genes. Yellow rust races capable of overcoming *Yr5* have been reported in India, China, Australia, and Turkey (Tekin et al. 2021; Zhang et al. 2022). Therefore, the need to release new resistant varieties, especially those with durable, non-race specific resistance, is urgent before new races emerge to overcome these genes (Sharma-Poudyal et al. 2020).

Race-specific resistance mechanisms that activate a hypersensitive immune response and visible development of necrotic lesions only at the point of pathogen infection is typical (Balint-Kurti 2019). Mutations affecting genes in the programmed cell death pathway, rather than NLRs can also create lesions that form without pathogen exposure, leading to the ‘lesion mimic’ phenotype, long known in maize (Hoisington et al. 1982) and other grasses (McGrann et al. 2015) and ‘flecking’ phenotype associated with fungal resistance in crops (McGrann et al. 2015; Olukolu et al. 2016). Lesion mimic mutants have provided invaluable insight into mechanisms of plant immunity and programmed cell death (Moeder and Yoshioka 2008) and their phenotypes are often temperature-dependent (Bruggeman et al. 2015). Cloned genes that give these phenotypes include several alleles of the maize *Rp1* (resistance to *Puccinia sorghi*) locus (Hu et al. 1996; Collins et al. 1999), and of these *Rp1-D21* forms spontaneous lesions suppressed by temperatures above 30 °C (Negeri et al. 2013). A similar suppression of lesion phenotype has been described in the rice mutant, *white and lesion mimic leaf1*, encoding a lumazine synthase in the riboflavin pathway (Hu et al. 2021), which is expressive at 20 °C but suppressed at 28 °C (Chen et al. 2018a). Another mechanism by which autoimmune leaf lesions can form is by combining NLR resistance proteins with non-coadapted host proteins. An example of this is spontaneous lesion formation in interspecific hybrid tomato leaves (Rooney et al. 2005). EMS mutagenesis of multiple antagonistic resistance genes could spawn lesion-mimic phenotypes by creating similarly maladapted immunity proteins, if co-expressed and co-localized.

Here, to leverage existing Kronos resources, we describe enhanced fungal resistance mutants identified using a forward genetic screen of previously mutagenized and sequenced lines (Krasileva et al. 2017) and augment these with newly customized Kronos-specific SNP mapping in an improved Kronos assembly. Two thousand lines from an ethyl methanesulfonate (EMS)-mutagenized tetraploid elite wheat *Triticum turgidum* subsp. *durum* cv ‘Kronos’ population have been characterized by exome capture and sequencing (Uauy et al. 2009; Krasileva et al. 2017; Zhang et al. 2023b). Mutant seed is available from several seed banks (Krasileva et al. 2017), and mutations in the population are incorporated into multiple plant genome browsers, such as GrainGenes (https://wheat.pw.usda.gov), Ensemble (https://plants.ensembl.org/Triticum_aestivum) and from the Dubcovsky Lab at UC Davis (https://dubcovskylab.ucdavis.edu/). Many applied breeding (Mo et al. 2018; Harrington et al. 2019; Li et al. 2021; Zhang et al. 2023a) and basic research programs (Schilling et al. 2020; Wang et al. 2020b; Corredor-Moreno et al. 2022) have published data from analyzing these mutations, highlighting their continued value to the community.

Our forward genetic screen preferentially identified mutations that prevent stripe rust sporulation, thereby reducing available field inoculum. These mutations complement the existing moderate adult plant resistance in Kronos (Chen 2023) and some are effective against other important fungal diseases including leaf rust and. We have increased the utility of the EMS lines by improving the accuracy of SNP mapping in the newly described Kronos enhanced disease resistance (EDR) lines and have shown the utility of our forward screen by mapping the single major resistance locus found in mutant line Kr620. These valuable new durum resistance alleles combined with an improved genome, further the utility of Kronos as a future model tetraploid wheat for genetic studies.

## Results

### Sixteen EDR lines persistently resistant to stripe rust

**Fig. 1:**
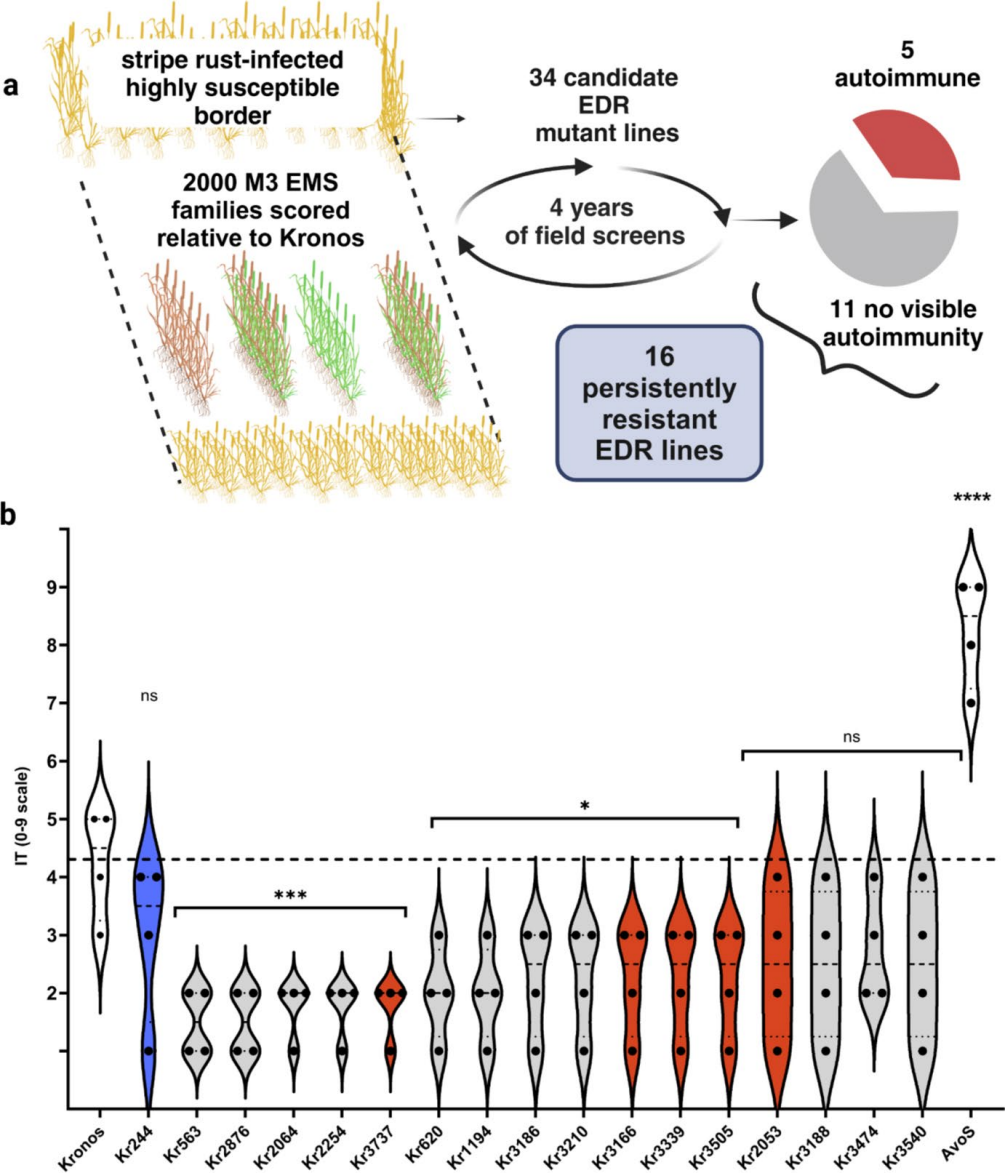
Sixteen persistently resistant Kronos stripe rust mutants. **a** Identification of stripe rust enhanced disease resistance (EDR) lines. Over two growing seasons, 2,000 EMS-mutagenized M3 lines were planted in 30 cm rows with susceptible border rows and were selected on their ability to prevent pathogen sporulation, compared to wild-type progenitor Kronos. **b** The performance of Kronos mutants against stripe rust in adult plants. Sixteen EDR lines are more resistant than the progenitor variety, Kronos (Kr0), to rust races over four years at the adult stage. Each dot represents a year of field testing conducted in 2014, 2019, 2020 and 2021 in Davis, California, USA. All presented mutant lines had an average Infection Type (IT) score of less than 3 and prevented pathogen sporulation. AvoS was the susceptible check variety. ITs of all plants are scored on the 0–9 scale (McNeal et al. 1971). Control lines (white) plus an EDR line with no macroscopic cell death under pathogen challenge (blue) are included for comparison with the autoactive lesioned lines (red) and non-autoactive lines (grey). Dotted line indicates Kronos median value of 4.25 in the adult stage experiment and 7.75 in the seedling stage experiment. Means of EDR lines were compared to Kr0 mean using one-way ANOVA, followed by Dunnett’s multiple comparison test. *, significant at *p* = 0.05; **, significant at *p* = 0.01; *** significant at *p* = 0.001; ****, significant at *p* < 0.0001; ns, non-significant

**Fig. 2:**
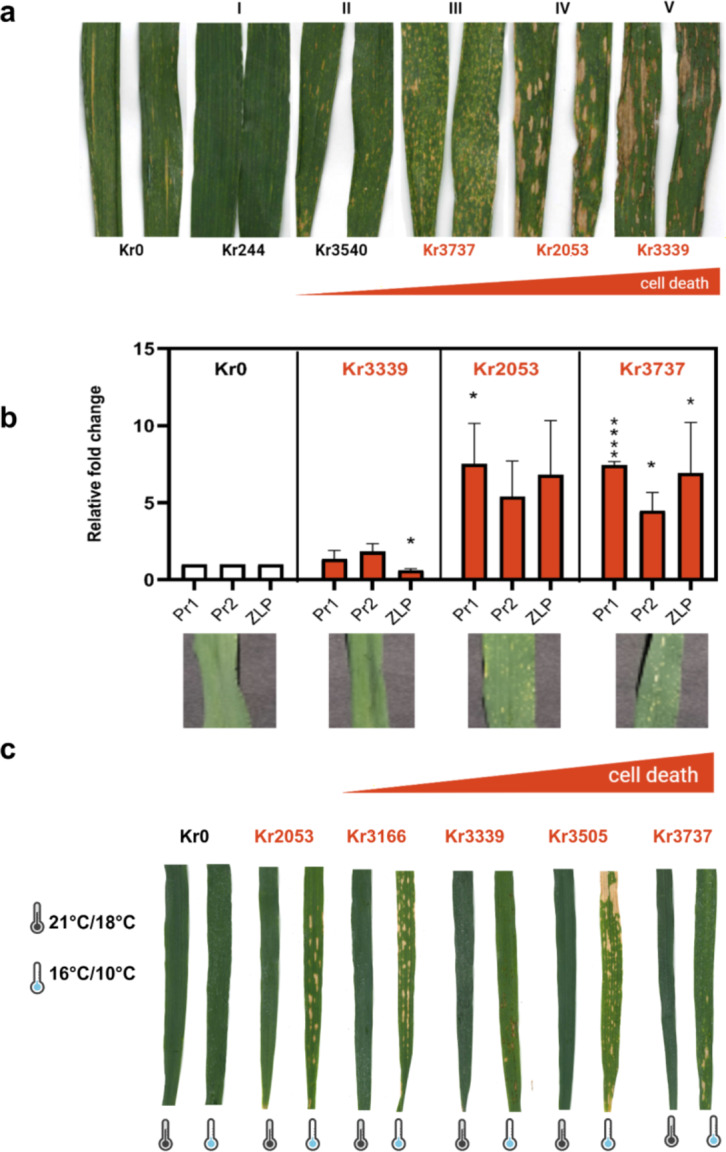
Characterization of autoimmune lines and their temperature dependency. **a** Excised flag leaves of Kronos (Kr0) and five observed phenotypic categories (I-V) of stripe rust-exposed, field grown enhanced disease resistance (EDR) mutants. Kr244, a non-autoimmune “green” EDR mutant, is included for comparison to representative mutants Kr3540, Kr3737, Kr2053 and Kr3339. **b** Relative fold change of rust-inducible genes *Pr1*, *Pr2* and *Zlp* correlates with autoactive lesion formation in growth chamber grown lesion mimic mutants. Means were compared using Holm-Šídák adjusted *p*-values (α = 0.05), error bars denote standard error. **c** Autoactive lesioned EDR mutants display cool temperature-enhanced cell death in the absence of the stripe rust pathogen. About 10% of putative EDR lines grown in fungicide-treated field plots in 2014 formed lesions beginning at growth stages Z37-Z39 (Zadok 1974). This phenotype was confirmed to be spontaneous and enhanced by cool temperatures, by growing the lines under normal greenhouse conditions (21 °C day/18 °C night; 16 h photoperiod) and in a cool growth chamber (16 °C day/10 °C night; 16 h photoperiod), respectively

To identify EDR lines with adult stage resistance to stripe rust, we conducted a forward genetic screen of 2,000 independent Kronos EMS-mutagenized M3 families (Uauy et al. 2009; Krasileva et al. 2017) that were screened in spring growing seasons in Davis, California. Half of the mutants were grown from 2012 to 2013 and the other half from 2013 to 2014. Field-collected *Pst* urediniospores from the previous season were used to inoculate highly susceptible border plants (Fig. 1a) to provide high pathogen pressure for effective plant resistance screening compared to wildtype Kronos progenitor controls. Wild type Kronos has intermediate stripe rust resistance at the adult plant stage and forms necrotic lesions with a light amount of rust sporulation. Based on 0 to 9 infection type (IT) scale (McNeal et al. 1971), ITs of 0 to 3 indicate that the pathogen did not form uredinia. Wild type Kronos had an average IT of 4 (ranging from 3 to 5) over both screening years with visible stripe rust uredinia appearing in necrotic stripes occupying ~ 10% of the flag leaf area. Thirty-four candidate M3 lines that prevented *Pst* sporulation were initially selected. M4 heads were collected from these 34 candidate lines and bulked for seed for future screening. M5, M7, M8 and M9 lines were grown again and scored for stripe rust resistance (Table S1) over four years. We defined each individual EDR line as having persistent resistance if its average IT was less than 3 in the four years 2014 and 2019 to 2021 and ITs in each year did not exceed 4 (Table S1). Of the 34 candidate EDR mutant lines originally identified by the screen as having ITs of 0—3 in 2013 (Table S2), 16 lines met the threshold of persistently resistant, and of these five had autoimmune lesion mimic phenotypes (Figs. 1 and 2). Of these 16 lines, five lines with an IT of no more than 2 and seven lines with an IT no more than 3 were significantly more resistant (*p* < 0.01, and *p* < 0.05, respectively) to stripe rust than Kronos at the adult stage. The other four lines with an IT of no more than 4 were not significantly different but had an IT lower than Kronos (Fig. 1b) by ordinary one-way ANOVA (*F* = 10.08; *p* < 0.05). Since the original disease screen of EMS mutants was conducted on adult plants, most EDR lines were similarly susceptible as Kronos at the seedling stage. An exception was Kr3186 which showed high seedling resistance to race PSTv-37 in greenhouse trials (Fig. S2).

### Characterization of autoimmune lines and their temperature dependency

Plants can be disease resistant without obvious lesions, but lesion formation indicates the activation of the hypersensitive response which may occur with or without pathogen exposure. Therefore, here, we evaluated resistance phenotypes through two metrics: stripe rust proliferation using an IT scale and prevalence of cell death lesions using a subjective visual classification. In addition to differences in ITs and successful uredinia formation, the pathogen-challenged flag leaves of EDR mutant lines showed a spectrum of cell-death response from no macroscopic cell death lesions to lesions covering more than half of the leaf (Fig. 2a; Fig. S1). Lesion initiation and lesion propagation/expansion, are the two characteristics used in describing and distinguishing these mutants (Walbot et al. 1983; Bruggeman et al. 2015). In our rust nursery in Davis, CA, USA the mutants fell into five major phenotypic classes (Fig. 2a; Fig. S1): I: no macroscopic cell death (*n = *1; Kr244), II: moderate cell death that covered up to 30% of the leaf surface (*n = *13; Kr3540 is shown), III: extensive cell death with many tiny lesions that speckled 80% or more of the leaf (*n = *1; Kr3737), IV: extensive cell death due to large oval lesions at the leaf tip and medium lesions in the proximal half of the leaf (*n = *1; Kr2053) and V: extensive cell death due to large, striped coalesced lesions that began in the leaf center (*n = *1; Kr3339). Within the 34 candidate EDR lines, only Kr244 lacked visible lesions. However, Kr244 fared progressively worse and did not meet the persistently resistant criteria of having a four year average IT of less than 3.

To determine which of the EDR lines were autoimmune as opposed to gain of pathogen recognition mutants, we grew plants in the absence of pathogens and observed lesion formation in five lines (Kr2053, Kr3166, Kr3339, Kr3505, and Kr3737) (Fig. 2c; Fig. S1a). The eleven EDR lines that formed lesions only in the presence of the pathogen all fell into phenotypic category II, while autoimmune lines had variation in lesion pattern and size (Fig. 2; Fig. S1). In rust-exposed field conditions, autoimmune Kr3737 (category III) was high in lesion initiation, having many pinprick-sized lesions that gave the leaves a speckled appearance, but low in lesion expansion. Whereas Kr3339 (V) had fewer but quite expansive lesions, Kr2053 (IV) was intermediate in its expression of both components. Thus, Kr3737, Kr2053, and Kr3339 comprised a phenotypic series of autoimmune-initiated cell death mutants (Fig. 2a). To explore the relationship between lesion formation and stripe rust resistance in the autoactive lines, we compared expression levels of three *Pst*-inducible genes, *Pr1*, *Pr2*, and a *Zeamatin-like protein* (*Zlp*) gene. Levels of the pathogenesis-related gene expression roughly correlated with the severity of lesion formation (Fig. 2b), indicating that these autoactive lines ectopically express *Pst*-inducible genes during spontaneous lesion formation.

Autoimmunity is often observed to be temperature dependent (Freh et al. 2022), with increasing lesion formation in low temperatures (Fu et al. 2009; Negeri et al. 2013; Chen et al. 2018a). The original screen and all follow-up California trials were conducted in Davis, CA, USA (38.536318°, −121.797405°) in *Pst*-inoculated plots without fungicide treatment, which allowed us to observe pathogen-dependent phenotypes at relatively high temperatures. The average daily temperature in Davis, CA in April of 2013 and 2014 was 17 °C with an average low of 9 °C and an average high of 24.9 °C (Nelsen et al. 2021). By contrast, the Kronos mutant population grown in June of 2015 at the John Innes Centre Field Station, Norwich, UK (52.631371°, 1.1791853°) in a fungicide-treated plot had the average daily temperature of 14.2 °C, an average low of 8.6 °C and an average high of 19.8 °C. From the original candidate list of 34 resistant lines (Table S2), nine autoimmune M4 lines were identified as having lesions or chlorosis under the cooler conditions in Norwich. For confirmation of these phenotypes, the candidate autoimmune lines and Kronos were grown in growth chambers under cool conditions (16 °C/10 °C day/night 16/8 h) and greenhouse conditions (21 °C/18 °C day/night (± 2 °C) 16/8 h) without rust pressure, and the sensitivity of the phenotype to cool temperatures was confirmed. Eight of the nine originally identified autoimmune lines, from the originally identified 34 EDR candidate lines, had more pronounced visible lesions under cooler (16 °C/10 °C day/night) temperatures. Kr456 did not form lesions under either tested condition (Fig. S1b). Uredinia germination and penetration of wheat stomata by the *Pst* germ tube is optimal at cool temperatures between 7 −12 °C (Sharma-Poudyal et al. 2014). Therefore, low-temperature activation of immune responses during the *Pst* infection interval could be beneficial and lead to enhanced resistance; our nighttime temperature of 10 °C sought to address if autoimmunity would be triggered under a temperature regime that promoted pathogen infection.

### Autoimmune EDR stripe rust mutants showed a possible all-stage trade-off to foliar necrotrophic pathogens but not to FHB

**Fig. 3:**
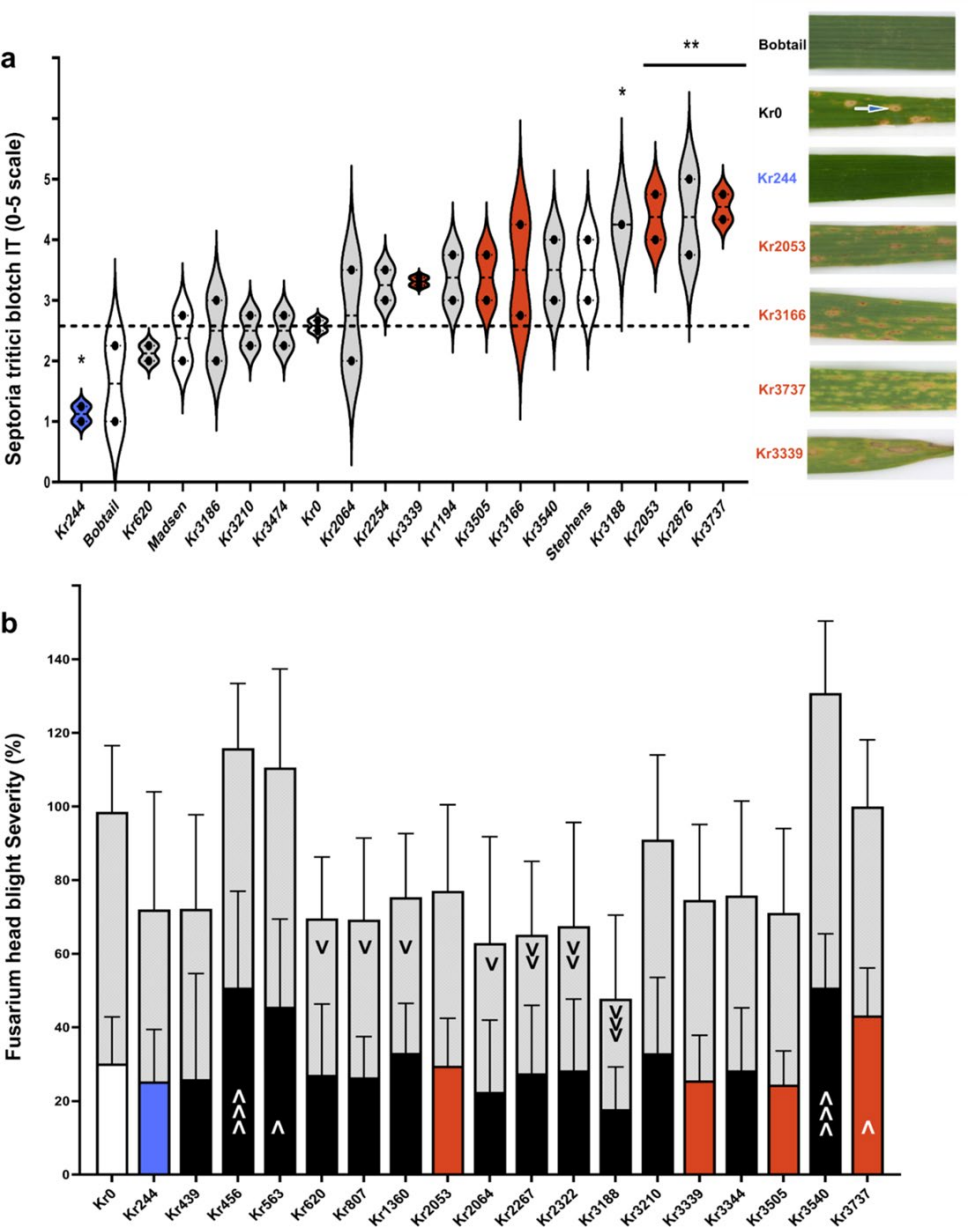
Response of autoactive lesion mimic enhanced disease resistance mutants (EDR) to necrotrophic pathogens. **a** (Left) Violin plot of ranked Septoria tritici blotch (STB) response (1–5 scale) of EDR lines shows trend that autoimmune lesioned lines have higher Infection Type (IT) scores than non-autoimmune lines. Each dot represents a year of field testing in 2021 and 2022 in Corvallis, OR. Means were compared using ANOVA followed by uncorrected Fisher’s Least Significant Difference (LSD). Control lines (white) plus an EDR line with no macroscopic cell death under pathogen challenge (blue) are included for comparison with the autoactive lesioned lines (red) and the non-autoimmune lines (grey). (Right) Excised flag leaves collected in STB nursery. Bobtail (resistant check), Kr0 with STB diagnostic black pycnidia (white arrow), Kr244 (blue) and four autoimmune lesion mimic plants (red). Dotted line indicates Kronos median value. **b** Control lines (white) plus an EDR line with no macroscopic cell death under pathogen challenge (blue) are included for comparison with the autoactive lesioned lines (red). Means of EDR lines were compared to Kr0 mean using nested one-way ANOVA multiple comparisons, and uncorrected Fisher’s LSD. *, significant at* p* = 0.05; **, significant at *p* = 0.01; *** significant at *p* = 0.001; ****, significant at *p* = 0.0001; ns, non-significant. **b** Fusarium head blight (FHB) severity (%) at 14 days post inoculation (dpi) (lower bars) and 21 dpi (upper gray bars) are shown. Significant differences are shown as “v” for less than or “upside-down v” for more than Kronos. Having one symbol indicates significance by ordinary one-way ANOVA, followed by uncorrected Fisher’s LSD, at the *p* = 0.05 level, two symbols indicate significance at the *p* = 0.01 level and three symbols indicate significance at the *p* = 0.001 level. Control lines (white) plus an EDR line with no macroscopic cell death under pathogen challenge (blue) are included for comparison with the autoactive lesioned lines (red) and the non-autoimmune lines (black)

Cell death lesions associated with hypersensitive responses are an effective immune strategy against biotrophic pathogens, but may increase plant susceptibility to necrotrophic pathogens that feed on dead tissue (McCombe et al. 2022). We examined whether the 16 EDR lines have differential adult responses to other pathogens and are more susceptible to a pathogen with a necrotrophic growth stage. These 16 lines were grown in a field highly infected with the *Septoria tritici* blotch (STB) pathogen, *Zymoseptoria tritici,* in Corvallis, Oregon, in replicated trials in 2021 (M8) and 2022 (M9). The EDR lines were sown in October to expose them to fall ascospore infections and subsequent infections via conidia in winter and spring. Spring wheat varieties, such as Kronos, typically survive the mild winters of Corvallis. The EDR lines and Kronos wild type were compared with the locally adapted winter cultivars Bobtail, Madsen, and Stephens, which are moderately resistant, moderately susceptible, and susceptible to STB, respectively. Scoring for STB IT was on a subjective 1 (little or no disease) to 5 (highly diseased) scale (Fig. S3a and Fig. S4). Kr244, which has little or no macroscopic cell death with *Pst* inoculation and is not autoimmune (Fig. 2a), performed better than Kronos in the STB nursery as shown by their average ranked scores over both years (Fig. 3a). Kronos showed characteristic pycnidial formation (Fig. 3a, white and blue arrow). Four lines performed worse than Kronos, and two of these have an autoimmune lesioned phenotype that is enhanced by cool temperatures (Fig. 2c and Fig. S1), which are typical of the early Oregon wheat growing season (during October—June). Adult leaves of these lines have leaves with necrotic tissue patches, potentially favored by the STB pathogen. All the mean STB scores for the autoimmune lines were higher than that of Kronos (Fig. 3a; Table S2).

We also assayed the EDR lines for response to another adult stage-infecting hemibiotrophic/necrotrophic pathogen, *Fusarium graminearum,* which causes fusarium head blight (FHB) of wheat, in greenhouse experiments. The 16 persistently stripe rust resistant EDR lines showed differential responses to *F. graminearum* inoculation both at 14 dpi (days post-inoculation) and at 21 dpi. Unlike their responses to the foliar diseases (STB, leaf rust and tan spot) which tended to be worse in mutants with autoimmune leaf necrosis (Fig. 3a, Fig. S2b, Fig. S4), three of the four tested autoimmune mutants were not significantly different from Kronos (Fig. 3b). At 14 dpi, the average FHB severity for susceptible Kronos was 30.2%. At 21 dpi, Kronos exhibited an average severity of 68.3%. Kr3188 showed high resistance at 21 dpi, but at 14 dpi, Kr3188 was not significantly different from Kronos. At the 21 dpi, two lines Kr2267 and Kr2322 had significantly lower severity at *p* < 0.01 and four lines (Kr628, Kr2267, Kr2322, and Kr3188) had significantly lower severity at *p* < 0.05. Four EDR lines, Kr456, Kr563, Kr3540 and Kr3737 had significantly higher severity all at 14 dpi (Fig. 3b). Altogether, our results showed that autoimmune lines tended to fare worse when exposed to STB. Interestingly, there was no strong positive or negative correlation between stripe rust resistance and FHB resistance despite varying responses of the EDR lies to FHB.

### Response of sixteen EDR lines to other fungal pathogens at the seedling stage

To assess if the resistance in the 16 mutant lines was all-stage and broad spectrum as well as persistent at the adult stage we performed seedling tests. When seedlings were challenged with the stripe rust pathogen *P. striiformis* f. sp. *tritici* PSTv-37, leaf rust pathogen *Puccinia triticina* race BBBQD, and powdery mildew pathogen, *Blumeria graminis* f. sp. *tritici,* Kr2053 and Kr3737 were not statistically different from Kronos (Fig. S2a, b, c, respectively). Notably, Kronos, Kr244, Kr2053 and Kr3737 were all resistant to race BBBQD (Fig. 3b). To further investigate possible enhanced susceptibility of the autoimmune lines to necrotrophic pathogens, we assayed their seedling response to the tan spot pathogen, *Pyrenophora tritici-repentis* and additional races (MNPSD and MPPSD) of the leaf rust pathogen, *P. triticina*. The autoimmune lines Kr3737 and Kr2053 had higher tan spot severity (%) and higher leaf rust IT than Kronos or Kr244 (Fig. 3b and d), even before the lesion phenotype was visibly expressed. This suggests that even at the seedling stage, autoimmunity may be active and compromise leaf defenses, conferring enhanced susceptibility to tan spot in these two mutants.

### Autoactive lesioned EDR mutants do not show reduced thousand kernel weight

To assess possible grain mass penalties due to autoimmunity or other mechanisms of enhanced resistance, we measured thousand kernel weights (TKW) in the EDR lines in our stripe rust trials. Most EDR lines had indistinguishable TKW values from Kronos (Fig. S5), except for three lines that had TKWs significantly higher than Kronos (one-way ANOVA, *p* = 0.05). Two lines Kr2053 and Kr3339 formed lesions with and without the rust pathogen, suggesting that in the presence of this pathogen, these lines are not inherently at disadvantage due to defense priming nor ectopic localized cell death lesions.

### Re-mapping of EDR line exome data to new Kronos long-read assembly

A chromosome-level assembly of the Kronos genome has recently become available (Seong et al. 2023). To support discovery of mutations responsible for EDR phenotypes, we remapped existing exome capture data of our 16 durably resistant lines onto this reference genome and identified single nucleotide polymorphisms (SNPs) with the MAPS pipeline (Table S9a) (Henry et al. 2014; Krasileva et al. 2017). We excluded two lines, Kr2027 and Kr2067, from our analysis due to missing data.

**Fig. 4:**
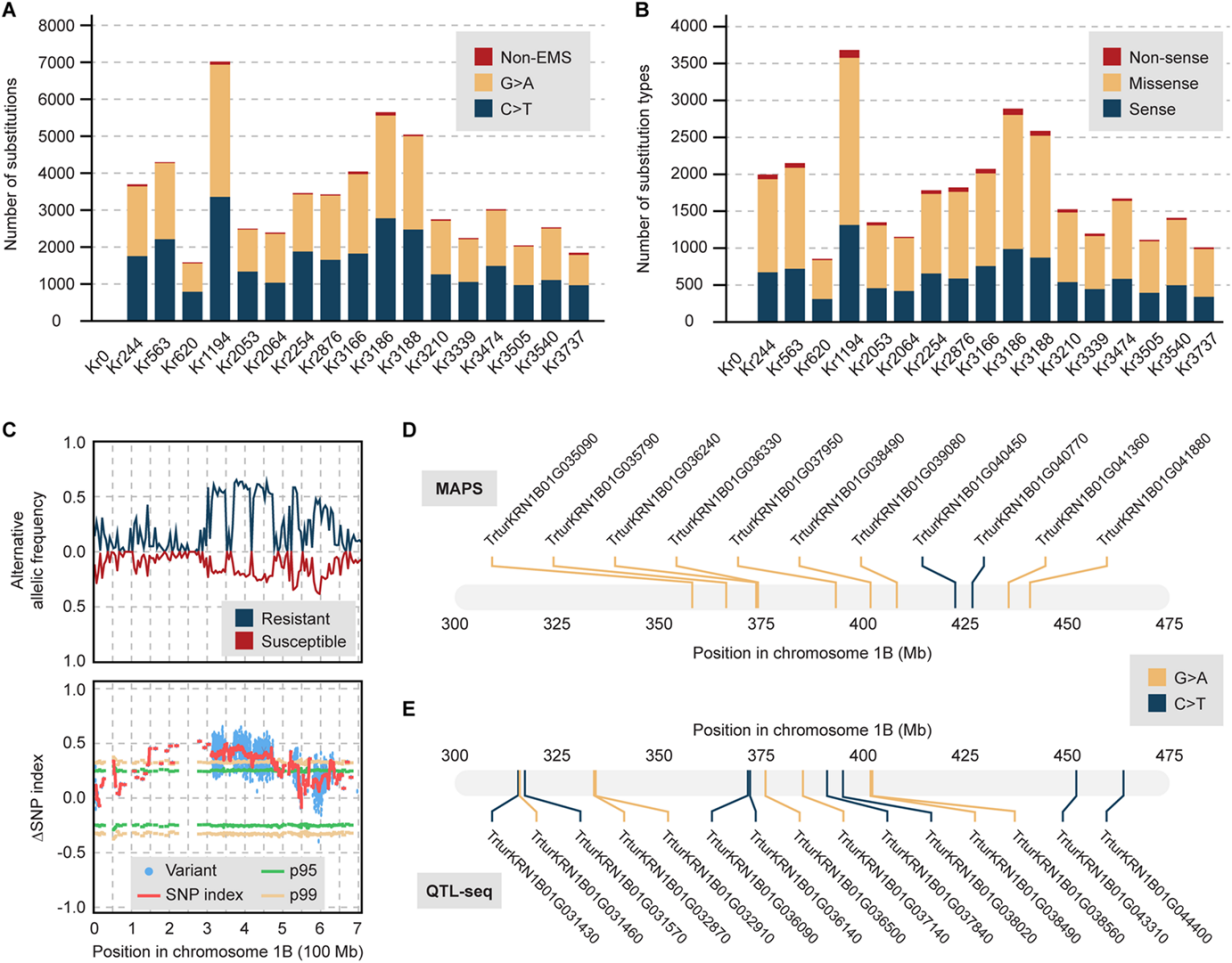
Identified substitutions in EDR lines and their impacts on proteins. **a** The number of substitutions identified with the MAPS pipeline from EDR lines and non-mutagenized control line Kr0. The mutations are colored to indicate non-EMS type mutations, and two EMS-type mutations (G to A and C to T). **b** The mutation effects of identified substitutions in coding sequences. **c** Allelic frequencies across chromosome 1B in 32 resistant and 13 susceptible F2 plants, generated from resistant Kr620 and susceptible Kronos (Kr0). (Top) a comparison of frequencies of detected alternative alleles across chromosome 1B in the resistant and susceptible bulks. The surveyed variants include all types of mutations detected in at least one resistant individual absent in wild type Kronos. (Bottom) a distribution of mean ∆SNP index calculated by QTL-seq. Blue dots indicate the presence of detected alternative variants, including both EMS-type and non-EMS-type mutations. ∆SNP index corresponds to the differences in frequency of alternative alleles in the resistant and susceptible bulks. Green and yellow lines correspond to confidence intervals at 95% and 99% of simulated ∆SNP index. **d** EMS-type mutations detected in resistant parent Kr620 that introduced missense mutations in the annotated coding sequences. **e** A subset of EMS-type mutations detected to be significant in the resistant bulk by QTL-seq. Among 74 EMS-type transitions that impacted the coding sequences, 15 SNPs are shown, as they had ∆SNP indexes greater than 0.5 and were detected as significant in 26 or more resistant F2 plants. These SNPs all introduced missense mutations in the annotated genes

To discern high-confidence mutations, our initial criteria required a minimum coverage of five mutant reads for heterozygous (HetMC) mutations and three for homozygous (HomMC) mutations, adhering to the methodology in the previous study (Krasileva et al. 2017). At this stringency, the MAPS pipeline identified only two substitutions from a non-mutagenized control line (Kr0), indicating no essential variation between the reference genome and the control line (Fig. 4a). Analysis of 32 EDR lines revealed 89,120 substitutions with 88,343 (99.1%) characterized as EMS-type transitions (Table S9a). These numbers surpass those reported using the hexaploid Chinese Spring genome as a reference (International Wheat Genome Sequencing Consortium (IWGSC) 2014; Krasileva et al. 2017). To maximize the number of detected mutations while maintaining the targeted EMS-type mutation rate of 98%, we adjusted the stringency of HomMC and HetMC as in the most recent study (Zhang et al. 2023b). We could identify 14,692 additional EMS-type mutations from the 32 EDR lines, averaging 3,220 total EMS-type mutations per line (Fig. 4a and Fig. S6; Table S9b).

We examined effects of the identified mutations on each EDR line, using 69,808 high-confidence genes (Seong et al. 2023). For each gene, we chose the annotated transcript producing the longest protein product. On average, 77% of all identified mutations impacted annotated genic regions, including coding sequences, splicing regions, introns and untranslated regions (Fig. S7). Approximately 7% of mutations were located in potential regulatory regions near genes, while 16% in intergenic regions. On average, mutations affected 1,654 genes per line, introducing 596 synonymous, 1,065 missense, and 46 nonsense mutations in coding sequences (Fig. 4b; Table S9c).

To identify potential gene candidates for the autoimmune mutants, we investigated mutations within orthologs of known lesion mimic mutants (LMMs), suppressors of LMMs, and *Yr* gene products (Table 1; Tables S10a-c) (Fu et al. 2009; Krattinger et al. 2009; Moore et al. 2015; Marchal et al. 2018; Zhang et al. 2019; Wang et al. 2020a; Klymiuk et al. 2020). In the five lines—Kr2053, Kr3166, Kr3505, Kr3737 and Kr3339—which exhibited distinct lesions in the absence of pathogens, we detected 22 missense mutations in 21 orthologs of LMMs. Kr2053 and Kr3339 both have allelic missense mutations in the Kronos ortholog of *Arabidopsis* candidate gene nonsense-mediated decay factor UPF1 (Table 1). Across the 32 EDR mutant lines, we identified 221 missense mutations and 5 nonsense mutations in 134 orthologs (Table S10b). Linkage testing and genetic complementation are needed to determine the causal loci in each lesion mimic mutant line, but this information can be used for prioritizing potential gene candidates.

**Table 1:** Substitutions within coding sequences of orthologs of LMMs

	Proteins in Kronos					Reference	
Mutant line	ID	Pos	Ref	Mut	Putative ortholog in Chinese Spring	*A.t* or *O.s* ID	Gene name
Kr2053	**TrturKRN1 A01G038710.1**	**397**	**R**	**K**	**TraesCS1 A02G259600.1**	**AT5G48380**	**BIR1**
Kr2053	**TrturKRN1 A01G039980.1**	**118**	**P**	**S**	**TraesCS1 A02G270000.1**	**Os10 g0195600**	**SPL18**
Kr2053	**TrturKRN2 A01G034020.4**	**1170**	**P**	**S**	**TraesCS2 A02G228000.1**	**AT5G47010**	**UPF1**
Kr2053	**TrturKRN2B01G044330.1**	**961**	**G**	**S**	**TraesCS2B02G258400.1**	**Os07 g0203700**	**SPL5**
Kr2053	**TrturKRN6 A01G001770.2**	**200**	**A**	**V**	**TraesCS6 A02G006400.1**	**AT1G55490**	**LEN1**
Kr2053	**TrturKRN7B01G031430.2**	**274**	**G**	**W**	**TraesCS7B02G201000.1**	**AT4G01370**	**MAPK4**
Kr3166	**TrturKRN2 A01G062630.1**	**145**	**P**	**S**	**TraesCS2 A02G437600.1**	**Os04 g0601800**	**WSP1**
Kr3166	**TrturKRN4B01G030810.1**	**13**	**P**	**L**	**TraesCS4B02G188200.1**	**AT4G33680**	**AGD2**
Kr3166	**TrturKRN6 A01G004900.1**	**416**	**G**	**D**	**TraesCS6 A02G024300.1**	**Os03 g0364400**	**BBS1**
Kr3166	**TrturKRN6 A01G038280.1**	**383**	**G**	**E**	**TraesCS6 A02G244000.1**	**AT5G51290**	**ACD5**
Kr3166	**TrturKRN7 A01G005700.5**	**343**	**G**	**C**	**TraesCS7 A02G036200.2**	**AT1G55490**	**LEN1**
Kr3339	**TrturKRN1B01G046720.1**	**70**	**D**	**N**	**TraesCS1B02G269900.1**	**AT5G48380**	**BIR1**
Kr3339	**TrturKRN2 A01G033500.2**	**514**	**T**	**I**	**TraesCS2 A02G231900.3**	**AT1G02120**	**VAD1**
Kr3339	**TrturKRN2 A01G034020.4**	**584**	**E**	**K**	**TraesCS2 A02G228000.1**	**AT5G47010**	**UPF1**
Kr3339	**TrturKRN5B01G027370.1**	**818**	**P**	**L**	**TraesCS5B02G160100.1**	**AT5G19400**	**SMG7**
Kr3339	**TrturKRN6B01G057640.2**	**408**	**G**	**D**	**TraesCS6B02G358000.3**	**AT5G64930**	**CPR5**
Kr3505	**TrturKRN1 A01G012390.2**	**358**	**V**	**I**	**TraesCS1 A02G065700.2**	**AT5G60410**	**SIZ1**
Kr3505	**TrturKRN1B01G052190.1**	**335**	**A**	**T**	**TraesCS1B02G307200.1.cds1**	**AT5G58430**	**EXO70B1**
Kr3505	**TrturKRN3 A01G047940.1**	**558**	**G**	**D**	**TraesCS3 A02G348200.1.cds1**	**AT5G58430**	**EXO70B1**
Kr3505	**TrturKRN5 A01G024990.1**	**53**	**P**	**S**	**TraesCS5 A02G175200.1**	**AT3G14110**	**FLU**
Kr3505	**TrturKRN5 A01G029260.1**	**147**	**S**	**F**	**TraesCS5 A02G205900.1**	**AT3G25540**	**LOH1**
Kr3737	**TrturKRN2B01G073290.1**	**653**	**A**	**T**	**TraesCS2B02G465300.1**	**Os01 g0116600**	**SPL33**

Mutant genes inducing lesion mimic phenotypes were identified in *Arabidopsis thaliana* (*A. t*) and *Oryza sativa* (*O. s*). Their orthologs in Kronos were investigated in the five EDR lines exhibiting autoactive lesion phenotypes and listed if missense mutations are present. No nonsense mutations were identified in the surveyed orthologs of all lesion mimic mutant genes

### Kr620 stripe rust resistance locus maps to chromosome 1B

Given the consistently robust resistance phenotype observed in Kr620 and that it yielded the most F3 seed, this mutant line was prioritized for the identification of stripe rust resistance loci (Table S1; Table S2). We generated exome-capture data from genomic DNA of 32 resistant and 13 susceptible F2 lines that were phenotyped as F3 families. Comparative analysis of alternative allele frequencies between resistant and susceptible bulks revealed a potential linkage on chromosome 1B within a 175 Mb interval, spanning from 300 to 475 Mb (Fig. 4C and S8). This interval showed higher alternative allele frequencies in the resistant pools compared to the susceptible pools. Using QTL-seq (Sugihara et al. 2022), we calculated the ∆SNP index, which reflects the significant difference in allele frequency between resistant and susceptible bulks (Fig. 4C and S8). The analysis consistently indicated a single locus on chromosome 1B as the source of resistance.

Examination of SNPs with the MAPS pipeline revealed 1,593 high-confidence EMS-type mutations in the M2 generation of resistant parent Kr620 (Fig. 4A). Among these, 117 SNPs were mapped to chromosome 1B, with 27 SNPs located within the 300 to 475 Mb interval. Notably, 11 SNPs introduced missense mutations in 11 different genes, but no nonsense mutations were detected (Fig. 4D; Table S11). The MAPS pipeline is designed to identify high-confidence EMS-type mutations unique to Kr620 that are absent in other compared Kronos mutant libraries (Krasileva et al. 2017). To ensure no mutations were overlooked by the MAPS pipeline, we also analyzed EMS-type mutations detected by QTL-seq in the F2 plants (Fig. 4E). This analysis uncovered 73 high-confidence missense mutations and one nonsense mutation in a protein of unknown function (TrturKRN1B01G034980). However, this nonsense mutation only affected three terminal amino acids. Most of these mutations were present in 25 or more individuals within the 32 resistant pools and distributed along the putative genomic loci responsible for resistance. These 74 mutations provide a highly reduced set of priority alleles to link the Kr620 resistance phenotype to a causative gene.

## Materials & methods

### Trials for adult biotrophic fungal infection response

#### Identification of stripe rust EDR lines and evaluation over multiple growing seasons

The initial forward genetic screening of an EMS mutant population of the wheat variety Kronos for *Pst* resistance was conducted in the fields at the UC Davis Field Station in Davis, California during spring of 2013 and 2014. In each of these two seasons 1,000 independent M3 families were evaluated. In all field assessments, each family or genotype was planted in a single 1 m long row, spaced 30 cm apart. To ensure sufficient inoculation pressure, these plots were surrounded by a highly *Pst-*susceptible dwarf CIMMYT breeding line, D6301. At plant growth stage (GS) 40–49 (booting) (Zadoks et al. 1974), the plots were inoculated with mixed races of *Pst* urediniospores collected from the same wheat fields the previous field season. *Pst* IT was scored on a 0–9 scale (McNeal et al. 1971) 2 to 4 times per season, separated by 7 to 10 days. In both years, non-mutagenized Kronos had an IT of 4 to 5. This intermediate resistance allowed for the identification of mutant families that displayed enhanced resistance. Once sporulation occurred on wild-type Kronos flag leaves, the field was observed at least twice for families that had plants with ITs lower Kronos. Mutants that did not display any visible sporulation were identified as candidate EDR lines. Single tillers of plants displaying deviant phenotypes were labeled, and the corresponding spike was harvested at maturity for subsequent evaluation. For evaluation over multiple growing seasons, seeds of the EDR lines were planted in 1 m long rows and inoculated as in the initial mutant screen described above. The EDR lines and Kronos were grown with identical spacing as in their original screening trials, but in triplicate randomized blocks.

#### Chamber validation of adult stripe rust EDR phenotypes

EDR lines with strong resistance phenotypes were included in validation experiments in controlled environment growth chambers and inoculated with *Pst*. To ensure a fresh and robust inoculation pressure, the highly *Pst*-susceptible lines Avocet S and Fielder were grown and inoculated with spores collected from the field in advance of planting the experiment. The experimental plants were inoculated at mid-tillering phase and again after flag leaf emergence. Freshly collected spores were mixed with technical grade talcum powder and applied to plants that had been misted with deionized water and kept in a dark dew chamber at 10 °C for 24 h. The temperature conditions of the chamber were then set to gradually increase from a nighttime temperature of 4 °C to 18 °C during the days with a photoperiod of 16 h. Pustules of *Pst* began to appear 14 days after inoculation and phenotypes were recorded 25 to 30 days after the inoculation.

#### High-throughput assessment of thousand kernel weight (TKW) under stripe rust exposure

For TKW assessment, 200–300 kernels of each EDR line and Kronos of each of three biological replicates were averaged over the three years, 2020 to 2022 in Davis, CA. Kernels were distributed evenly in a (11.3 cm × 13.1 cm) box, weighed and photographed with a 9 th generation iPad camera (model A2197). The number of seeds in each photograph was determined using the CountSeeds App (Version 2.229.0 Build 6) with the Wheat Seed template (ID: 393 Version: 003) and the average weight per kernel was determined. This result was multiplied by 1,000 to give the estimated TKW. Three technical replications were made of each biological replicate, without replacement.

### Identification and validation of autoimmune EDR mutants

Nine autoimmune mutant lines were originally identified as having lesions in a fungicide-treated plot (rust-free) in Norwich, UK, 2015 (fungicide schedule listed in Table S2b). For confirmation of these phenotypes, this subset of lines and Kronos were grown in 3 inch pots using Supersoil #4 supplemented with Osmocote 14–14-14 (N-P-K) at 3.3 g/L fertilizer and grown in a growth chamber under cool conditions (16 °C/10 °C day/night 16/8 h). Eight of the nine identified lines had lesions under these conditions, but none had lesions under standard greenhouse conditions (21 °C/18 °C day/night 16/8 h).

### Real-time quantitative PCR (RT-qPCR) analysis

Kronos, Kr2053, Kr3339, and Kr3737 were grown in 8″ plastic pots (1.4L) in LM-6 soil (Lambert, Canada) in the greenhouse at 22 °C/16 °C day/night with a 16 h photoperiod. Flag leaves (Zadok’s GS 55) were collected in three replicates from each accession and immediately frozen in liquid nitrogen. Total RNA was extracted using TRIzol reagent (Invitrogen, cat. no. 15596018) according to the manufacturer’s instructions. DNA contamination was removed by treating total RNA samples with DNase I (Roche, Cat. No. 04716728001), followed by phenol–chloroform purification and precipitation. First-strand cDNA was synthesized from 500 ng of total RNA using the SuperScript™ IV first-strand synthesis kit (Invitrogen, Cat. No. 18091200). RT-qPCR was then performed in four technical replicates for each sample with the PowerUp™ SYBR™ green master mix (Applied Biosystems, Cat. No. A25741) on the QuantStudio™ 5 Real-Time PCR System, 384-well (Applied Biosystems, Cat. No. A28140) with the following program: UDG activation at 50℃ for 2 min, activation (Dual-Lock™ DNA polymerase) at 95℃ for 2 min, followed by 40 cycles of denaturing at 95℃ for 15 s, annealing/extension at 60℃ for one min. Relative expression levels of *Pr1-1–1* (*TraesCS5B02G181500*), *Pr-2-1c* (*TraesCS3 A02G483000*) and *Zlp* (*TraesCS4 A02G29630*) were calculated using the comparative cycle threshold (2^−ΔΔCt^) method (Livak and Schmittgen 2001) with normalization to the internal control gene *Actin*. Raw data and calculations are provided in Table S3. All primers used for RT-qPCR are shown in Table S8.

### Adult plant screens of EDR lines for necrotrophic pathogen response

#### Field trials for STB response

In our 2020 pre-trial of Kronos and a smaller set of tester EDR lines, we had aphid-transmitted barley yellow dwarf virus pressure. Hence, in 2021 and 2022 we applied Cruiser Maxx Vibrance Cereals (Syngenta) at a rate of 0.48 mL per g of seed and included all available lines and controls. The EDR lines were grown in four replicates at the naturally infected STB plot in Hyslop Field (44.63284°, −123.19315°) in Corvallis, OR, in a randomized complete block design. STB infection type was scored on a subjective 1 (resistant) to 5 (susceptible) scale (Fig. S3b) using moderately resistant, moderately susceptible, and susceptible check varieties Bobtail, Madsen and Stephens, respectively.

#### Greenhouse tests of FHB severity

Kronos wild type was used as a control and EDR lines were tested using five plants per genotype planted in a randomized complete block design in 4 inch × 4 inch pots in the greenhouse, in the following soil mix: Sungro professional mix SS#1, Metromix 830 and Surface Greens grade (2:2:1). Temperature conditions were 23–25 °C during daytime and 16–18 °C at night with 16 h of light. Highly virulent *F. graminearum* isolate GZ3639 (Desjardins et al. 1997) was used as the inoculum. To generate macroconidia, 2–3 fungal mycelial culture plugs from Potato Dextrose Agar were introduced into Mung bean broth (MBB). Macroconidia were counted using a hemocytometer, and an inoculum was created by diluting the culture to 1 × 105 spores/ml using sterile water. Inoculations were conducted at the pre-anthesis stage, approximately two days before anthers emerged from the spikes. The 10 th and 11 th spikelets, counted from the base of the spikes, were marked. Ten microliters of inoculum were pipetted into one floret of each spikelet between the lemma and the palea, taking care to avoid injury to the other parts of the florets. Following this, the spikes were enveloped in moisture-saturated plastic bags for 72 h to create a high-humidity environment conducive to optimal fungal growth (Chhabra et al. 2021b). Bleached spikelets were counted from the point of inoculation downward, and % severity was calculated by dividing bleached spikelets by total number of spikelets beneath the inoculated spikelet (10) at 14 dpi and 21 dpi.

### Seedling screens of EDR lines to multiple fungal pathogens

#### Stripe rust

Five to seven plants of each EDR line or Kronos, and the susceptible check Avocet S were grown in a 7 cm cubic pot in our custom soil mix (30% peat moss, 10% perlite, 15% sand, 15% Sunshine potting soil, 20% vermiculite and 10% water) supplemented with Osmocote 14–14-14 (N-P-K) fertilizer at 3.3 g/L. The reactions of those lines to stripe rust were evaluated with the current predominant race PSTv-37, virulent to *Yr6*, *Yr7*, *Yr8*, *Yr9*, *Yr17*, *Yr27*, *Yr43*, *Yr44*, *YrTr1*, and *YrExp2*, and is avirulent to *Yr1*, *Yr5*, *Yr10*, *Yr15*, *Yr24*, *Yr32*, *YrSP*, and *Yr76* (Wan and Chen 2014)*.* Fresh urediniospores of PSTv-37 were mixed with Novec 7100 fluid (3 M Inc., St. Paul, MN, USA) at a ratio of 10 mg/mL and evenly sprayed on wheat plants at 2-leaf stage with an atomizing sprayer. The plants were incubated at 10 °C in a dark dew chamber for 24 h, and subsequently, the plants were moved to a controlled growth chamber with the temperature gradually increasing from 4 °C at 4:00 am to 22 °C at 4:00 pm and then decreasing to 4 °C with 16 h light/8 h dark for stripe rust development. The IT score of each EDR line was recorded 18 days after inoculation using a 0 to 9 IT scale (Line and Qayoum 1991) of which IT 0 to 3 was considered resistant, IT 4 to 6 was considered intermediate, and IT 7 to 9 was considered susceptible.

#### Leaf rust

### *P. triticina* races MNPSD and MPPSD inoculation

Two seeds of each line were sown in a single cell of 72-celled trays filled with a commercial ‘Redi-Earth’ soil (Sun Gro, Bellevue, WA, USA). Plants were raised in a rust-free greenhouse at 20 °C/18 °C (day/night) and a 16 h photoperiod. Jack’s classic® all-purpose (20–20-20) (N-P-K) fertilizer was applied to the seedlings once a week. The EDR lines were planted in 4 replications. Leaf rust susceptible checks, including Thatcher, TAM110, Chisolm and Danne, and a resistant check, Thatcher-*Lr19*, were included in the test (Table S6). Seedlings were inoculated at the 2nd leaf stage with a suspension of urediniospores comprised of a mixture of races collected from Oklahoma in 2021 and 2022 (primarily *P. triticina* races MNPSD and MPPSD) and light mineral oil (Soltrol 170; 1 mg of urediniospores per 1 mL of oil) using an inoculator pressurized by an air pump. The oil was allowed to evaporate from the leaves for at least 30 min before incubating in dark at 20 °C for about 18 h and relative humidity of 100%. Following incubation, plants were transferred to the greenhouse at 20 °C/18 °C (day/night) and a 16 h photoperiod.

Leaf rust ITs were assessed at 12 dpi on the first leaves of seedlings using a 0-to-4 scale (Stakman et al. 1962) where IT 0 = no visible symptom, ‘;’ = hypersensitive flecks, 1 = small uredinia with necrosis, 2 = small- to medium-size uredinia surrounded by chlorosis, 3 = medium-size uredinia with no chlorosis or necrosis, and 4 = large uredinia with no necrosis or chlorosis. Larger and smaller uredinia than expected for each IT were designated with + and −, respectively. Lines showing ITs of 0 to 2 + were considered resistant, while 3 and 4 scores were considered susceptible (Long and Kolmer 1989; McIntosh et al. 1995). For data analysis, the 0-to-4 scale was converted to a linearized 0-to-9 scale as previously described (Borlaug Global Rust Initiative 2011; Zhang et al. 2014). Briefly, 0, 1-, 1, 1 +, 2-,2, 2 +, 3-, 3 and 3 + are decoded as 0, 1, 2, 3, 4, 5, 6, 7, 8 and 9, respectively. Ratings of 0 to 6 were classified as resistant IT and 7 to 9 were considered as susceptible IT.

### *P. triticina* race BBBQD inoculation

For the leaf rust screening, wheat seedlings at the two-leaf stage were evaluated for their reactions to BBBQD in the biosafety level 2 (BSL 2) facility at Dalrymple Research Greenhouse Complex, North Dakota Agricultural Experiment Station (AES), Fargo. Briefly, 3 to 4 seedlings per EDR line along with susceptible checks were used for phenotypic screening for BBBQD with 3 technical replicates. Plants were grown in trays containing PRO-MIX LP-15 (Premier Tech Horticulture) sterilized soil mix and maintained in a rust-free greenhouse growth room set to 22 °C/18 °C (day/night) with 16 h/8 h light/dark photoperiod. At two-leaf stage, the seedlings were inoculated with fresh urediniospores suspended in SOLTROL-170 mineral oil (Phillips Petroleum) at a final concentration of 10^5^ spores per ml using an inoculator pressurized by an air pump. RL6089 and Little Club were used as susceptible checks. The inoculated seedlings were placed in a dark dew chamber at 20 °C overnight and then transferred back to the growth room.

The ITs were scored at about 12 to 14 dpi, using the 0–4 scale (Stakman et al. 1962). For each IT,'+‘or’-'was used to represent variations from the predominant type. A'/'was used for separating the heterogeneous IT scores between leaves with the most prevalent IT listed first. For plants with different ITs within leaves, a range of ITs was recorded with the most predominant IT listed first. The IT scores were converted to a 0–9 linearized scale referred to as infection response (IR) (Borlaug Global Rust Initiative 2011; Zhang et al. 2014). Plants with linearized IR scores of 0–4 were considered as highly resistant, 5–6 as moderately resistant, and 7–9 as susceptible.

#### Powdery mildew

Seeds of each line were sown in 8.25 in. × 1.5 in. diameter plastic cones containing a commercial ‘Ready-Earth’ soil (Sun Gro, Bellevue, WA, USA). The lines were planted in 4 replicates with two plants each. Plants were raised in the greenhouse at 20 °C/18 °C (day/night) and a 16 h photoperiod. Seedlings were inoculated at the 2nd leaf stage by gently shaking heavily infected plants of Jagger, infected with Oklahoma field isolates of *Blumeria graminis* f. sp. *tritici* over seedlings of the tested lines. Approximately 7 to 10 days after inoculation, ITs on the plants were rated on a 0–5 scale with 0 being the most resistant reaction and 5 being the most susceptible reaction. Plants having scores of 0–2 were considered resistant, plants having a score of 3 were considered intermediate, and plants having a score of 4 −5 were considered susceptible. Powdery mildew susceptible check variety Jagger was included as a control.

#### Tan spot

Seeds of each line were sown in 8.25 in. × 1.5 in. diameter plastic cones containing a commercial Redi-Earth’soil (Sun Gro, Bellevue, WA, USA). The lines were planted in 8 reps with two plants in each rep. Plants were raised in a growth chamber at 20 °C/18 °C (day/night) with a 16 h photoperiod. Tan spot susceptible checks ‘Billings’and TAM 105, an intermediate check ‘Karl 92’, and a resistant check ‘Red Chief’ were included in the test. Wheat seedlings were inoculated at the third leaf stage (1 st and 2nd leaves fully expanded) using an atomizer (DeVilbiss Co., Somerset, PA, USA) with a conidial suspension (2,000 conidia per ml). An Oklahoma isolate of *P. tritici-repentis* Race1 was used for the inoculation. About one hour after inoculation, when conidia adhered to dried leaves, seedlings were placed in a mist chamber that provided near 100% relative humidity for 48 h. Plants were then placed in a growth chamber at 20 °C/18 °C (day/night) with a 16 h photoperiod. One week after inoculation, 1 st and 2nd leaves were rated to determine percent leaf area infected by tan spot. For further analysis, we considered severity on the 2nd leaves.

### Remapping EDR mutant exome capture data to new Kronos long-read assembly

The exome capture data for a non-mutagenized control (Kr0) and 32 EDR lines were downloaded from the National Center for Biotechnology Information (NCBI) (Table S9a) (Krasileva et al. 2017) and mapped to the Kronos reference genome (Seong et al. 2023; https://zenodo.org/records/10215402). Two EDR lines, Kr2027 and Kr2067, were excluded in this analysis as their data were unavailable. The paired-end datasets were first trimmed and filtered to remove sequencing adapters and low-quality reads (quality scores below 20) with fastp v0.23.2 (Chen et al. 2018b). The processed reads were mapped to the Kronos genome with bwa aln v0.7.17-r1188 (Li 2013). The alignments were sorted with samtools v1.15.1 (Li et al. 2009) and duplicates were removed with picard v3.0.0 (https://github.com/broadinstitute/picard). These filtered alignments were processed with the MAPS pipeline (Henry et al. 2014). A minimum mapping quality of 20 was required for alignments to be processed. A minimum of 20 libraries were needed to identify valid mapping positions. HetMinPer was set to 15, and the following pairs of HomMC and HetMC were chosen: (2, 3), (3, 2), (3, 4), (3, 5), (4, 3), (5, 3) and (6, 4). Regions of residual genetic heterogeneity were removed. For each mutant individual library, the parameter producing a maximum number of mutations with a EMS-type mutation rate of 98% or higher was selected (Zhang et al. 2023a, b).

### Mapping of a stripe rust resistance locus in Kr620

To localize the stripe rust resistance locus in Kr620, a resistant Kr620 plant was crossed with pollen from the susceptible Kr0 progenitor line to generate F1 seeds, which were advanced to produce F2 seeds. Genomic DNA was extracted from leaf tissue collected from individual F2 plants. F3 families, derived from single F2 heads, were cultivated during the 2016 field season in Davis, California. Phenotyping of the F3 families for stripe rust resistance identified 20 putatively homozygous susceptible families, 78 putative heterozygous families, and 43 putatively resistant families, indicating segregation distortion or category misassignment (Chi-square, *p* = 0.106). Resistant families were over-represented, suggesting that Kr620 has a dominant resistance locus. However, other mutations or field conditions made identifying susceptible families unreliable. In a modified bulked segregant analysis (BSA) (Michelmore et al. 1991) barcoded exome-capture libraries were prepared for each of 32 homozygous resistant and 13 homozygous susceptible F2 plants, as well as 3 wild type Kronos (Table S12). The samples were sequenced and processed using the exome data mapping workflow, with the exception that the alignment was performed with bwa mem v0.7.17-r1188. SNPs were called with GATK HaplotypeCaller v4.5.0 (McKenna et al. 2010). With bcftools v1.2.0 (Danecek et al. 2021), alternative variants identified in the resistant pool were selected based on a quality score threshold of above 30 and depth of coverage (DP) threshold greater than 10, provided these variants were absent in the wild type pool. Reference and alternative alleles at these positions were quantified with bam-readcount v0.8 and VAtools v5.1.1 (Khanna et al. 2021). To minimize noise, only sites where the average DP for both resistant and susceptible pools exceeded 20 were included for visualization. QTL-seq v2.2.4 was utilized to supplement the analysis (Takagi et al. 2013; Sugihara et al. 2022), directly quantifying SNP indexes from the pooled alignments from the wild type, resistant and susceptible pools. SNPs discussed in this study are EMS-type substitutions with ∆SNP index greater than p99 calculated by QTL-seq.

### Mutation effect analysis

The mutation effect in each EDR line was examined with snpEff v5.2a (Cingolani et al. 2012). All analyses relied on a high-confidence gene set that contains 69,808 genes, unless specified. The effect was analyzed on a transcript per gene that encodes the longest protein sequences.

### Orthology inference

The putative orthology was inferred with OrthoFinder v2.5.5 (Emms and Kelly 2019). Proteomes from six species were analyzed, including *Arabidopsis thaliana* (Araport11) (Cheng et al. 2017), *Oryza sativa* (IRGSP-1.0) (Kawahara et al. 2013), *Solanum lycopersicum* (ITAG 4.0) (Hosmani et al. 2019), *Triticum aestivum* (IWGSC from Ensembl Plants) (Bolser et al. 2016; International Wheat Genome Sequencing Consortium (IWGSC) 2018), *Zea mays* (NAM 5.0) (Woodhouse et al. 2021) and Kronos. Both high and low-confidence gene models were included for Kronos. Only the longest protein products per gene were used. OrthoFinder relied on Diamond v2.1.7 (Buchfink et al. 2015) for homology inference, MAFFT v7.525 (Katoh et al. 2002) for sequence alignments, and FastTree v2.1.11 (Price et al. 2010) for constructing phylogenetic trees. Orthogroups that included known LMMs, suppressors of LMMs and Yr gene products were identified. Orthology was categorized as one-to-many, many-to-one or many-to-many, based on the number of proteins from a reference species and Kronos. Typically, only a limited number of members were identified in most analyzed orthogroups for Kronos and common wheat. In more complex cases involving gene duplications and deletions, reciprocal best blast searches were employed to match putative orthologs between two species (Camacho et al. 2009). For Yr gene products, phylogenetic trees were also utilized to infer orthology.

### Statistical analysis

**Fig. 5:**
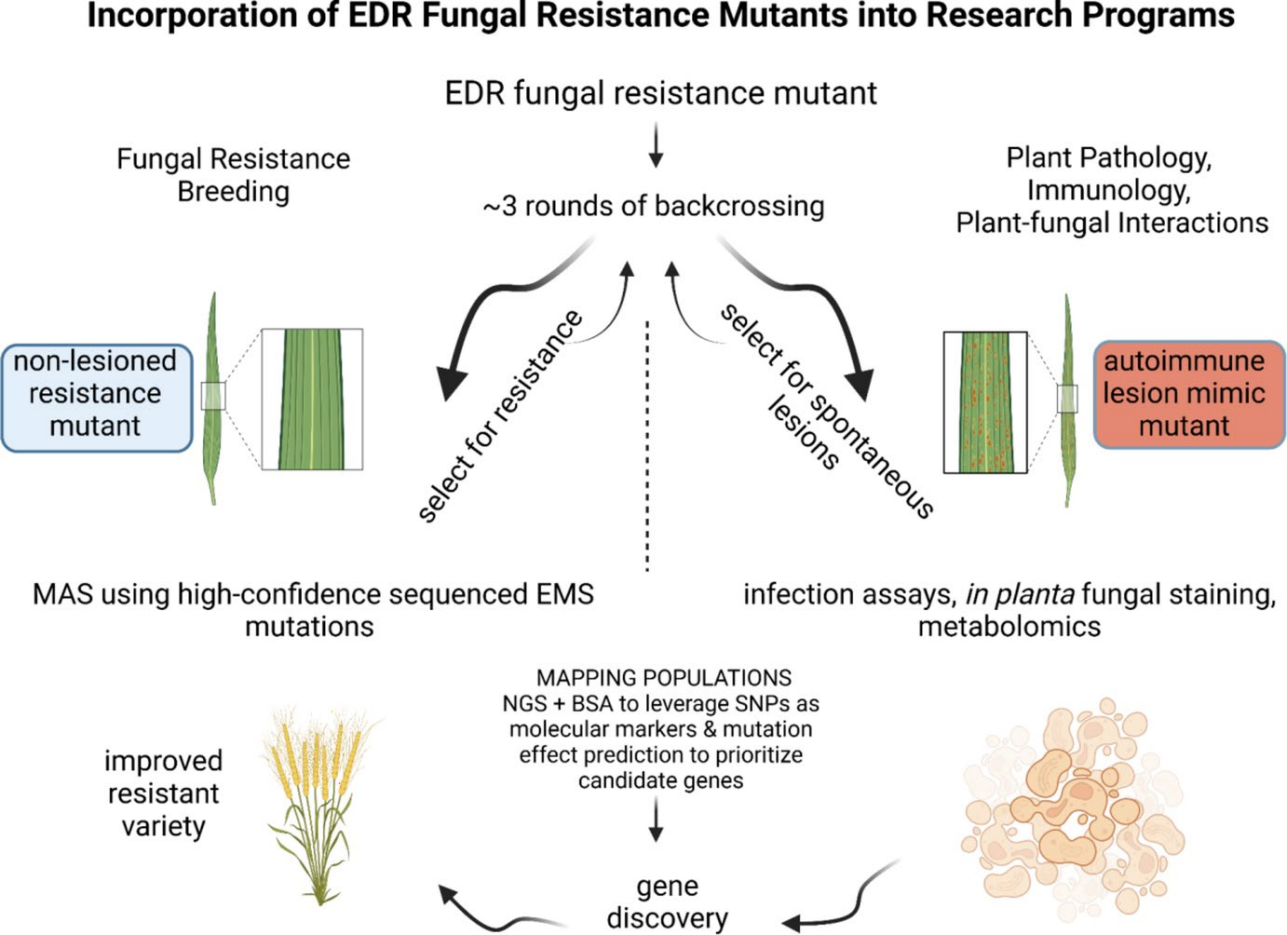
Applied breeding and basic research approaches using EDR lines with fungal resistance. The left flowchart depicts incorporation of EDR line alleles into resistance breeding programs. The right flowchart depicts incorporation of EDR line alleles into plant pathology, immunology, or plant-fungal interaction research programs

Graphs and graphics for Figs. 1, 2, 3 and 5 were generated using GraphPad Prism 10.2.3 for Windows, GraphPad Software, Boston, Massachusetts USA, www.graphpad.com and combined into figures at BioRender.com. Means were compared using ordinary one-way analysis of variance (followed by Dunnett’s multiple comparisons, for comparing two or more groups against a single control group, test where applicable) and Fisher’s LSD test or unpaired t-tests followed by Holm-Šídák method of multiple comparison adjustment, for the quantitative RT-PCR comparisons. Data and statistical analyses supporting figures are provided in the Supplementary Tables file (T_S3-S7). Figure 4 was created using Python v3.10.12 and the matplotlib 3.8.3 package of python.

## Discussion

Our search for new alleles for wheat fungal resistance breeding yielded 34 independent EMS-mutagenized Kronos EDR lines, 16 of which had persistent multi-year increased adult plant resistance to stripe rust. Similarly, Hussain et al. (2018) created a hexaploid wheat EMS population from cultivar NN-Gandgum-1, also moderately resistant to stripe rust, and found 20 EDR lines from 3,624 M2 plants with an efficiency of 0.55%. We obtained 0.8% efficiency in a Kronos mutagenized population, indicating that forward screens for stripe rust resistance in EMS wheat populations is an effective strategy. The screen yielded five phenotypic categories of lesion formation from none to severe. Five EDR lines had additional ‘flecking necrosis’ or lesion mimic phenotypes without the pathogen, indicating ectopic lesion formation (autoimmunity) that was enhanced in cooler temperatures. Such lines with perturbed regulation of lesion formation may provide insight into the regulation of immunity-induced cell death processes. The mutations can also be used in basic research programs to uncover mechanisms of plant-pathogen interaction and programmed cell death.

The EDR lines, which were originally selected for adult foliar resistance to the biotrophic stripe rust pathogen, but were subsequently tested against other fungal diseases, showed variable adult response to the necrotrophic FHB and STB pathogens, with a tendency of the autoimmune lesion mutants to fare worse against the foliar STB pathogen and having no obvious correlation against the FHB pathogen, which infects inflorescences. Seedlings of autoimmune EDR lines Kr2053 and Kr3737 outperformed Kronos when challenged with some leaf rust and tan spot isolates but not in response to Oklahoma isolates of powdery mildew. Tradeoffs in resistance against various fungal pathogens have been well described (Kliebenstein and Rowe 2008), and therefore we sought to characterize the EDR lines in reaction to diverse pathogens, in search of lines that combine multiple resistances without penalty. The stripe rust pathogen, *P. striiformis*, is an obligate biotroph requiring living tissue to sustain its growth (Voegele et al. 2009). In contrast, *Z. tritici* and *P. tritici-repentis* gain entry to host tissues by causing cell death and colonizing necrotic tissues after a variable latent period which may or may not be biotrophic (Steinberg 2015). The autoimmune lines tended to fare slightly worse than the other EDR lines under challenge by the latently necrotrophic foliar pathogen, *Z. tritici*, and the necrotrophic foliar pathogen, *P. tritici-repentis*, suggesting a biotrophic-necrotrophic trade-off, as has been previously reported (Spoel et al. 2007; He et al. 2022)*.* In our study, EDR line Kr244, with no macroscopic cell death performed better than Kronos against Septoria leaf blotch. Susceptibility of EDR autoimmune lesion mimics to necrotrophs was milder at the seedling stages, as expected, as lesions only appear after the juvenile phase. Additionally, since the EDR lines were phenotypically selected for high-temperature adult-plant resistance, it is possible that some lines carry all-stage resistance and that many solely carry adult resistance. Although the FHB pathogen, *F. graminearum*, is also a mostly necrotrophic pathogen (Kheiri et al. 2019), the autoimmune EDR lines performed similarly to Kronos in our trials. The EDR lines did show a differential response to FHB as was seen in an EMS population of hexaploid wheat, ‘Jagger’ (Chhabra et al. 2021a). We surmise that the lack of correlation between stripe rust resistance and FHB resistance may be because FHB is a disease of the inflorescence rather than a foliar disease. Our phenotypic selection for resistance to the foliar stripe rust disease may have favored leaf-specific fungal resistance mechanisms rather than resistance genes expressed in floral tissues. Answering these questions requires further investigation.

As expected, generally, EDR lines with autoimmune lesions performed more poorly in tests with necrotrophic pathogens than the non-autoimmune EDR lines, but interestingly, they did not have a reduction in TKW. It should be recognized that TKW is only one component of yield. Resistance versus yield tradeoffs have been previously documented (Draz et al. 2015; Olukolu et al. 2016) so comprehensive yield analyses should be done while incorporating EDR lines into breeding programs. Within each line, a combination of mutated and wild type alleles contributes to each phenotype, necessitating continued rounds of phenotypic selection for durable, quantitative resistance.

To showcase the utility of the EDR lines in fungal resistance breeding programs, we mapped a major stripe rust resistance locus in the Kr620 line to a defined interval using the recently released long-read genome of Kronos (Seong et al. 2023) and mapping by sequencing with bulk segregant analysis methods. We identified a 175 Mb interval on chromosome 1B and cataloged the EMS type mutations and their likelihood to cause protein changes within this region. Of note are 15 SNPs causing missense mutations that were detected as significant in 26 or more resistant F2 plants. While our bulked segregation analysis can aid identifying the locus conferring resistance, the current dataset may not fully resolve the genetic map. A limitation of exome capture sequencing is that it is dependent upon pre-designed baits, which were generated based on transcriptomic data from Kronos and several other species to target genic regions of the genome (Krasileva et al. 2017). It is possible that causative mutations reside in regulatory regions or genes within the identified genomic loci that were not included in the bait design. Further genetic mapping using custom markers identified by newly mapping the polymorphisms in the Kronos EDR lines, additional mutagenesis of candidate genes to generate revertant alleles, and/or targeted sequencing using baits specific to the the potential stripe rust resistance 1B interval would provide further resolution.

To highlight the resources presented here, we have made a schematic representation of two pipelines that may be used for research using the EDR lines (Fig. 5). The resistance breeding trajectory (left side) begins with an EDR line without visible autoimmune phenotypes. Because the mutational load in these lines is high, these mutants may carry quantitative resistance in which one major locus contributes to the resistance phenotype (as was found in Kr620 and is suggested by the seedling stripe rust resistance in Kr3186) or, in some lines, several alleles may contribute to resistance. Introgression of the alleles by backcrossing to elite durum varieties may be advantageous to reduce the likelihood of linkage drag of undesirable EMS mutations. The publicly available EMS mutations are readily available for molecular marker development to aid in transferring EDR germplasm into elite varieties for release. While selecting for adult-stage stripe rust resistance, alleles contributing to bacterial pathogen resistance or abiotic stresses may have been selected or the stripe rust resistance genes may be pleiotropic, giving multi-stress protection. Conversely, we present FHB and STB data because it indicates that some EDR lines have increased susceptibility to pathogens other than *P. striiformis* and this may be a consideration when breeding for geographical regions in which multiple diseases are prevalent.

A basic approach to inform plant pathology and plant–microbe interactions (right side) is presented too, beginning with the introgression of an autoimmune lesion mutant. Once in a uniform genetic background, it is possible to characterize and quantify the effects of the mutation by using microscopy, infection assays and metabolomics. Gene cloning is a possible outcome of either research approach and may lead to additional information for hypothesis testing.

These newly described durum EDR lesion mimic lines provide a resource for further study of mechanisms of plant cell death and potential hormone signaling in wheat. In dicots, salicylic acid (SA) and jasmonic acid (JA) signaling are antagonistic; SA is induced in response to biotrophs and JA is induced in response to necrotrophs and herbivores (Stout et al. 2006; Kliebenstein and Rowe 2008). However, in rice, which has endogenously high levels of SA, both hormone pathways converge to confer immunity through a common pathway that can be activated by either hormone and can confer resistance to pathogens regardless of lifestyle (Tamaoki et al. 2013). Interestingly, a recent report (Benke et al. 2022) showed that SA was not required for autoimmune lesion formation in the *Rp1-D21#4* allele. The autoimmune mutants described herein provide a tool to address such mechanistic questions as whether this aspect of the allele is truly rare, or if SA is never or only sometimes required for lesion formation.

Further experiments could characterize these mutants in-depth, by focusing on the morphological aspects of their cell death phenotypes as previously described (van Doorn et al. 2011). Transcriptomic analyses are also warranted, for example, we found that *Pr1*, *Pr2* and *Zlp* are induced in two of the three autoactive EDR lines we tested and seemed correlated with the severity of the lesion phenotype. This suggests that at least in a subset of EDR lines, the LMM phenotype is consistent with ectopic activation of known plant immune pathways. Table 1 is presented as a list of candidate genes for the autoimmune lines. For example, Kr3505 has non-allelic mutations in two Kronos orthologs of the *Arabidopsis* candidate exocyst subunit EXO70B1, and loss-of-function of mutations in this resistance gene result in spontaneous lesions (Stegmann et al. 2013). Further characterization of these mutants by transcriptomic and metabolomic analyses as well as identification of causative alleles would uncover lesion mimic pathways shared across plants.

Overall, we show that these elite durum wheat EMS lines have enhanced resistance to stripe rust and other fungal pathogens. The mutations in these lines have already been sequenced, are incorporated into genome browsers and include candidate mutations in previously known EDR genes with readily available seed stocks for reverse genetics studies and importantly, easy molecular marker development from catalogued linked polymorphic SNPs available for each line. We newly mapped these mutations to a recent high-quality long-read Kronos genome, and we expect this resource will greatly aid in the identification of causal variants leading to their deployment in both basic and applied research programs.

## Supplementary Information

Below is the link to the electronic supplementary material.

Supplementary file1 (PDF 7190 KB)

Supplementary file 2 (XLSX 469 KB)

## Data Availability

Exome capture data generated in this study for bulked segregation analysis of Kr620 is deposited under the NCBI: PRJNA1151137. All scripts and workflows used in this study are available at Github: https://github.com/krasileva-group/Kronos_EDR. The data generated in the re-analysis of exome capture data can be accessed through https://zenodo.org/records/11099763.
